# Targeting the Reconsolidation of Licit Drug Memories to Prevent Relapse: Focus on Alcohol and Nicotine

**DOI:** 10.3390/ijms22084090

**Published:** 2021-04-15

**Authors:** Segev Barak, Koral Goltseker

**Affiliations:** 1 School of Psychological Sciences, Tel Aviv University, Tel Aviv 69978, Israel; 2 The Sagol School of Neuroscience, Tel Aviv University, Tel Aviv 69978, Israel; 3 Zuckerman Mind Brain Behavior Institute, Columbia University, New York, NY 10027, USA

**Keywords:** memory reconsolidation, addiction, alcohol, nicotine, tobacco, relapse, animal models

## Abstract

Alcohol and nicotine are widely abused legal substances worldwide. Relapse to alcohol or tobacco seeking and consumption after abstinence is a major clinical challenge, and is often evoked by cue-induced craving. Therefore, disruption of the memory for the cue–drug association is expected to suppress relapse. Memories have been postulated to become labile shortly after their retrieval, during a “memory reconsolidation” process. Interference with the reconsolidation of drug-associated memories has been suggested as a possible strategy to reduce or even prevent cue-induced craving and relapse. Here, we surveyed the growing body of studies in animal models and in humans assessing the effectiveness of pharmacological or behavioral manipulations in reducing relapse by interfering with the reconsolidation of alcohol and nicotine/tobacco memories. Our review points to the potential of targeting the reconsolidation of these memories as a strategy to suppress relapse to alcohol drinking and tobacco smoking. However, we discuss several critical limitations and boundary conditions, which should be considered to improve the consistency and replicability in the field, and for development of an efficient reconsolidation-based relapse-prevention therapy.

## 1. Introduction

Alcohol and nicotine are the two most commonly abused legal substances, with the highest substance-attributable mortality rates (3.3 and 6 million people annually, respectively) [1,2]. Excessive alcohol and tobacco use can also have adverse social and economic effects on the individual and society as a whole [1]. Both alcohol drinkers and tobacco smokers often fail to maintain long-term abstinence, and relapse rates remain very high [3,4].

Relapse to drug abuse can be triggered by craving, induced by exposure to environmental cues that have been previously associated with the reinforcing properties of the drugs [5,6,7,8]. Thus, disruption or attenuation of the cue-drug memories is expected to reduce the cue-induced craving and relapse.

Current theories hold that the retrieval of consolidated memories induces their temporal destabilization, which is followed by their re-stabilization in a process termed “reconsolidation” [9,10]. Certain pharmacological manipulations following memory retrieval abolish the subsequent behavioral expression of the target memory, suggesting the disruption of the ongoing reconsolidation process [11,12]. Thus, the term “reconsolidation window” was proposed, implying that interference with memory reconsolidation during this 5 to 6 h window of opportunity can attenuate the retrieved memory [9,10].

In drug addiction research, interference with the reconsolidation process was shown to attenuate and even prevent relapse to drug seeking and intake [13,14,15,16,17]. Performance can also be impaired by introduction of a new competing learning shortly after the retrieval of the memory, viewed as the incorporation of new information into the retrieved memory trace [9,18,19,20,21,22,23,24,25].

Here, we reviewed studies investigating the reconsolidation of memories associated with alcohol or nicotine/tobacco smoking, in animal models and in human subjects. Our review indicates that alcohol- and nicotine-associated memories can potentially be disrupted by post-retrieval manipulations, possibly due to interference with their reconsolidation process, which leads to reduced relapse. However, as we describe here, multiple boundary conditions should be considered and controlled to leap further towards the development of an efficient reconsolidation-based relapse prevention therapy.

## 2. Alcohol

### 2.1. Pharmacological Interference with Alcohol-Memory Reconsolidation

To date, the pharmacological manipulations aimed to disrupt the reconsolidation of alcohol-associated memories have focused on protein synthesis inhibition [14,26,27], NMDA receptor blockade [26,28,29,30,31,32], and β-adrenergic receptor blockade [28,32,33,34,35,36].

#### 2.1.1. NMDA Receptor Blockade

Blockade of NMDA receptors was the first manipulation reported to interfere with the reconsolidation of alcohol-associated memories, as reflected by reduced alcohol-seeking behavior [26]. To this end, rats were trained to self-administer 10% alcohol, with olfactory and auditory cues to signal alcohol availability. Following 3 weeks of abstinence, rats were exposed for 5 min to the alcohol-associated cues and non-reinforced lever pressing to retrieve and reactivate alcohol-associated memories. The authors found that administration of the NMDA receptor antagonist MK-801 (0.1 mg/kg, ip) immediately following memory retrieval reduced the cue-induced increase in alcohol seeking by ~25%, as compared to vehicle-treated controls. In a repeated test conducted 7 days later, MK-801-treated rats showed, only a non-significant trend towards reduced alcohol seeking [26]. This study suggested that the alcohol-memory reconsolidation could be affected by inhibition of the NMDA receptor activity. In contrast, post-retrieval injection of the FDA-approved drug acamprosate (200 mg/kg, ip), a combined GABA agonist/NMDA antagonist used for alcohol use disorder treatment, failed to affect subsequent alcohol seeking [26].

The common protocol to target memory reconsolidation includes a single event of a pharmacological manipulation following memory retrieval. Wouda et al. [32] tested whether a repeated post-retrieval blockade of NMDA receptor-dependent signaling could disrupt alcohol seeking. They trained rats to self-administer 12% alcohol by performing a nose poke during the presentation of a light cue, and alcohol delivery was signaled by a tone. After 3 weeks of abstinence, the memory was retrieved by the presentation of the alcohol-associated cues only (light + tone, with the nose-poke holes covered). Immediately after the 20-min retrieval session, rats were injected with MK-801 (0.1 mg/kg) or saline, and were tested for alcohol-seeking 24 h later. This retrieval→treatment→test block was repeated 3 times, 6 days apart, resulting only in a trend towards reduction in alcohol-seeking in rats that received MK-801 after the memory retrieval [32].

Milton et al. [28] showed that the reconsolidation of alcohol-associated memories could be disrupted by the well-timed administration of an NMDA-receptor antagonist. First, they trained rats to lever-press for 10% alcohol in the presence of alcohol-predicting cues following a stimulus discrimination training. For memory retrieval, rats were repeatedly presented with the alcohol-predictive cues. Here, unlike in most reconsolidation studies, MK-801 (0.1 mg/kg, ip) was injected 30 min before, rather than after, the retrieval session. In a test 24 h later, non-reinforced operant responding was measured in the presence of the alcohol-predicting and non-predicting cues. Rats that received saline prior to memory retrieval increased lever-pressing during the presentation of the alcohol-paired cues, whereas rats that received MK-801 prior to memory retrieval responded similarly to the alcohol-paired and unpaired cues, suggesting that NMDA receptor blockade disrupted the reconsolidation of the cue-alcohol memories [28].

However, the same group also showed that NMDA-receptors blockade prior to memory retrieval does not always affect memory reconsolidation [30]. Thus, MK-801 administered before a short non-reinforced lever-responding session failed to impair alcohol seeking in rats. The authors concluded that operant (instrumental) memories [30] are more resistant to this manipulation following retrieval, compared with the Pavlovian memories targeted in their previous study [28]. Indeed, targeting the memories underlying operant behaviors via reconsolidation mechanisms has been particularly challenging [37,38], yet feasible with a thorough selection and adjustment of memory retrieval procedures [25,38,39,40,41].

Another NMDA receptor antagonist, memantine, was also tested for its effects on alcohol memory reconsolidation in an operant procedure [31]. Rats were trained to operantly self-administer alcohol, signaled by light and tone. After extensive training (15 sessions) and 2 days of abstinence, rats received a short training session (5 min), aimed to retrieve alcohol-associated memories. Memantine (20 mg/kg, ip) was injected twice: immediately after memory retrieval, and 4 h later. During a cue-induced alcohol-seeking test 24 h later, the animals that received memantine responded less on the alcohol-associated lever. Surprisingly, similar reduction in alcohol seeking was observed in rats that received memantine without prior memory retrieval, suggesting that memantine affected alcohol seeking 24 h after its administration, regardless of memory retrieval [31]. Therefore, it was impossible to conclude regarding its effects on alcohol memory reconsolidation. Notably, following extinction training, rats that received memantine after memory retrieval showed impaired reacquisition of the alcohol self-administration, compared to the animals that received saline. Arguably, this effect could also be interpreted as enhanced reacquisition of alcohol-lever responding in the rats that received saline after memory retrieval, similarly to other observations of memory strengthening following undisturbed memory retrieval and reconsolidation [42].

In humans, ketamine administration following the retrieval of the alcohol-associated memories in hazardous drinkers reduced the reinforcing effects of alcohol and long-term drinking levels, compared to ketamine or retrieval alone [29]. Blood concentrations of ketamine and its metabolites during the critical “reconsolidation window” predicted beneficial changes only following memory retrieval. These findings suggest that NMDA receptor blockade has the capacity to reduce alcohol relapse in humans, presumably by affecting reconsolidation mechanisms.

In summary, the attempts to impair the reconsolidation of the alcohol-associated memories by blockading the NMDA-receptors signaling yielded conflicting yet promising results. Apparently, a successful targeting of the alcohol memories that undergo NMDA receptor-dependent reconsolidation requires a carefully chosen set of temporal and procedural parameters. Furthermore, a close examination of the available data reveals that the NMDA receptor blockade manipulations had more pronounced effects on newer [28] rather than older memories [31,32]. Indeed, previous observations showed that older memories are more resistant to post-retrieval interference [43,44,45,46].

#### 2.1.2. β-Adrenergic Receptor Blockade

β-adrenergic receptors were among the first pharmacological systems studied for their involvement in memory reconsolidation [12,47,48]. In addition, there is a well-documented involvement of the noradrenergic system in psychostimulant addiction [49] and in cognition [50].

In a mouse conditioned place preference (CPP) paradigm, propranolol (10 or 30 mg/kg, ip) failed to affect the reconsolidation of alcohol-associated memories when given after their retrieval [35]. In an attempt to directly target the brain regions suggested to play a role in the maintenance of the alcohol-associated memories, Chesworth and Corbit [34] infused propranolol (2 µg per hemisphere) or vehicle into the basolateral amygdala (BLA) of rats trained to lever press for alcohol. Propranolol or vehicle infusion was preceded by a training session aimed to retrieve the alcohol-related memories. A day later, rats that received post-retrieval propranolol treatment showed reduced responding for alcohol in the first 6 trials. However, due to extinction in the control vehicle group, no effect was found in the subsequent 12 trials [34], thus limiting the conclusions about the effect of the intra-BLA blockade of the β-adrenergic receptors on memory reconsolidation.

Using an operant self-administration paradigm in rats, Milton et al. [28] showed that administration of propranolol (10 mg/kg, ip) 30 min before the retrieval of the alcohol-related memories did not affect alcohol-seeking behavior. Specifically, male rats learned to discriminate between alcohol-predicting and non-predicting cues when self-administering alcohol. They were then injected with propranolol or saline 30 min before the retrieval of the memory by a brief exposure to the alcohol-predicting cues. On the next day, both groups showed increased response in the presence of alcohol cues, indicating that propranolol did not disrupt the reconsolidation of the alcohol memories underlying the conditioned approach and motivation [28].

In another study, the same group showed that reconsolidation of certain aspects of alcohol-associated memories could be impaired by the β-adrenergic receptors blockade [33].Rats were first trained to nose-poke for an alcohol reward signaled by a light cue. During a subsequent second-order conditioning phase, rats acquired lever-pressing with the alcohol-associated light cue serving as a positive reinforcer. Treatment with propranolol (10 mg/kg, ip) prior to a memory retrieval session (via nose-poke-light only) disrupted the lever pressing in a test one day later [33]. These findings may suggest that β-adrenergic blockade interfered with the reconsolidation of the alcohol-associated memories. Notably, administration of the adrenergic prodrug dipivefrin (10 µg/kg, ip) before memory retrieval enhanced the capacity of the alcohol-associated light cue to act subsequently as a conditioned reinforcer, suggesting that the reconsolidation of specific aspects of alcohol memories can be bidirectionally modulated by reducing and enhancing central adrenergic signaling [33].

Interestingly, in another study that tested the effect of β-adrenergic blockade on alcohol-memory reconsolidation, alcohol-seeking behavior in rats was reduced only after two and three sessions of retrieval-propranolol treatment [32]. Therefore, it is possible that alcohol-memory reconsolidation can be impaired only by repeated blockade of the β-adrenergic receptors.

The efficacy of the repeated post-retrieval propranolol treatment was further demonstrated in human hazardous drinkers [36]. In a double-blind study, treatment-seeking adults diagnosed with substance dependence received double-blind propranolol or placebo on six occasions prior to reading a personalized script detailing a drug-using experience (memory retrieval). Consequently, self-reported craving intensity was reduced in the drinkers who received propranolol prior to alcohol-memory retrieval [36].

Taken together, these findings suggest that whereas the β-adrenergic blockade may work better following repeated retrieval-propranolol cycles, its efficacy in disrupting reactivated memories should be further characterized.

#### 2.1.3. Protein Synthesis Inhibition

Probably the strongest and most consistent finding in the memory reconsolidation studies is that the retrieved memories can be disrupted by inhibition of protein synthesis [9,10,16]. However, only a few studies tested this strategy in alcohol-associated memory reconsolidation.

The non-selective protein synthesis inhibitor anisomycin, administered intra-cerebroventricularly, was shown to disrupt alcohol seeking in an operant self-administration paradigm, when given after memory retrieval (a 5 min extinction session), and this effect was still seen 7 d later [26]. In another study, alcohol seeking and self-administration was similarly suppressed by injection of anisomycin into the central amygdala (CeA) prior to memory retrieval [14].

Additional evidence for the critical role of protein synthesis in the reconsolidation of alcohol-associated memories was provided by studying the involvement of the mammalian target of rapamycin complex 1 (mTORC1) pathway in the processing of these memories [14]. mTORC1 is a kinase that controls the translation of a subset of dendritic proteins, and as such, plays a role in rapid protein translation at the synapses, in synaptic plasticity, and in learning and memory functions [51,52]. In a series of experiments, Barak et al. [14] demonstrated that alcohol memories could be retrieved not only by the context and stimuli of the operant setting associated with alcohol, but also by the intrinsic sensory properties of alcohol per se (odor-taste cues). Specifically, after 7 weeks of drinking in a 2-bottle choice procedure followed by several weeks of operant self-administration training, rats were subjected to 10 days of abstinence. Then, alcohol memories were retrieved in two ways: by a brief (5 min) re-exposure to the operant context with an oral alcohol prime and with non-reinforced lever presses, or in the home cage, by short exposure (10 min) to the odor-taste cue of alcohol [14].

Retrieval of alcohol-associated memories induced activation of the mTORC1 pathway in the CeA, and in the orbitofrontal and prelimbic cortices, which led to increases in several synaptic proteins [14]. Prevention of these increases by post-retrieval systemic administration of the mTORC1 inhibitor rapamycin (20 mg/kg) disrupted the reconsolidation of alcohol-related memories, leading to long-lasting (14 days) suppression of relapse to alcohol seeking and consumption. Critically, this effect was present only when rapamycin was injected immediately, but not 5 h after the retrieval session, confirming that timing of mTORC1 inhibition is critical and should be conducted within the “reconsolidation window”. As the activation of mTORC1 following memory retrieval was localized to the CeA, rapamycin was locally administered into this brain region. Specifically, infusion of rapamycin into the CeA after memory retrieval prevented relapse to alcohol seeking and consumption, suggesting that it disrupted the reconsolidation of alcohol-related memories [14].

The disruptive effect of rapamycin on the reconsolidation of alcohol-memories was further confirmed by Lin et al. [27] who showed that systemic administration of rapamycin (10 mg/kg, ip) after re-exposure to the alcohol-paired environment decreased the expression of alcohol-CPP, and the effect lasted for up to 14 days and could not be reversed by a priming injection of alcohol [27].

### 2.2. Behavioral Interference with Alcohol-Memory Reconsolidation

The toxicity or side-effects of most relevant pharmacological amnesic agents [9,53] facilitated the development of behavioral interference with memory reconsolidation [24,40,41,54,55,56,57]. In this approach, a behavioral manipulation aimed to counteract the original maladaptive behavior is performed shortly after memory retrieval [21,41,55,58,59,60].

Using this approach, Cofresi et al. [57] showed that memory retrieval prior to extinction training reduced alcohol-seeking behavior, as compared to extinction with no memory retrieval. Rats were first trained to consume alcohol from a sipper presented into the training chamber only upon the onset of a visual cue. During the following 14 sessions of extinction training with an empty sipper, the retrieval group received a 1 h time-out in the home cage between the first two extinction trials, to retrieve alcohol-memories and initiate their reconsolidation. When tested two days later, rats that underwent extinction training during memory reconsolidation showed reduced spontaneous recovery and reinstatement of conditioned responses to alcohol-associated cues, suggesting that the retrieval-extinction procedure reduced relapse to alcohol seeking [57].

Surprisingly, Millan, Milligan-Saville and McNally [55] showed that alcohol-seeking behavior could be attenuated not only when memory retrieval occurs before an extinction session, but also when the retrieval occurred after extinction. Specifically, rats were trained to self-administer decarbonated beer in one context (context A), whereas extinction training was held in another, distinct context (B). Rats then received a 50 min extinction session, with an additional 10 min retrieval session given either 70 min before extinction (retrieval-extinction group) or after extinction (extinction-retrieval group). In a test conducted in the beer-associated context A, animals that underwent extinction before or after memory retrieval showed reduced context-induced reinstatement (renewal) of alcohol-seeking behavior, compared with no-retrieval controls [55]. Thus, while the retrieval-extinction findings could be interpreted as extinction occurring during the reconsolidation, leading to memory updating, the extinction-retrieval findings could not be interpreted in terms of reconsolidation. It is therefore possible that two shorter extinction sessions (50 min + 10 min regardless of their order) are more effective in reducing the conditioned response for alcohol, compared to a single extinction training (60 min, no retrieval group).

Noteworthy, the reduced response seen after extinction training typically recovers when tested for spontaneous recovery, renewal, and reinstatement [8]. In contrast, disruption of memory reconsolidation is expected to disrupt the memory; therefore, alcohol-seeking behavior should not re-emerge in these tests [9], allowing distinction between reconsolidation and extinction mechanisms [61]. However, the persistence of the decreased alcohol seeking was not compared between the retrieval-extinction, extinction retrieval, and no retrieval groups [55], limiting the interpretation of the findings. Nevertheless, Millan, Milligan-Saville and McNally [55] showed that retrieval-extinction facilitated the reacquisition of alcohol self-administration, emphasizing the limitation of this procedure in reducing alcohol seeking. Together, these findings suggest that the retrieval-extinction approach is not always effective in impairing the target memory and its behavioral expression, also see [62].

A related approach to reduce alcohol seeking via behavioral interference with memory reconsolidation employs aversive counterconditioning training following memory retrieval. In this approach, a cue, previously associated with the reinforcing effects of alcohol, is re-associated (counterconditioned) with aversive consequences [63]. Aversion therapy, based on counterconditioning, was shown to be more potent than extinction in suppressing relapse in animal models and humans studies [64,65], and to help alcohol drinkers to stay abstinent for a longer period [66]. However, its suppressive effect is temporary [67,68,69]. Similar to extinction, aversive counterconditioning is thought to lead to the formation of a new cue-aversion association that competes with the cue–alcohol association for behavioral expression [67,68]. Thus, the persistent cue–alcohol memory can recover, triggering craving and relapse.

We have recently demonstrated that relapse to alcohol seeking can be prevented by aversive counterconditioning, conducted during alcohol-memory reconsolidation, in both classical and operant learning paradigms [25]. Mice were first trained for alcohol-CPP. Next, the alcohol-associated context was counterconditioned with an aversive experience, a cold-water flooding [70], preceded or not by memory retrieval, i.e., a 3 min re-exposure to the alcohol context. In a following alcohol-primed test, alcohol-CPP was reinstated in the no-retrieval group, but not in the retrieval group, suggesting that aversive training disrupted the retrieved alcohol memory [25]. Moreover, mice from the retrieval-counterconditioning group avoided the alcohol context during the test [25], further suggesting that aversive information presented following memory retrieval can be integrated into the original memory, thus updating or replacing it [9,24,54,71].

Moreover, in contrast to the previous observation of the equivalent effects of retrieval-extinction and extinction-retrieval procedures on alcohol seeking [55], counterconditioning prevented the reinstatement of drug seeking only when applied after, but not before, memory retrieval [24], suggesting that the loss of drug seeking is mediated by memory reconsolidation mechanisms.

We further tested the efficacy of the retrieval-counterconditioning paradigm in an operant self-administration procedure that models relapse-like alcohol-related behaviors [72,73]. Specifically, rats were trained to self-administer alcohol in context A for 9 weeks, and then received punishment of lever-pressing with mild foot-shocks in a distinct context (context B) with or without prior 10 min exposure to the odor-taste cues in the home cages [25]. We found that without memory retrieval, or with memory retrieval given long before the punishment, rats showed renewal of alcohol seeking (i.e., non-reinforced lever pressing) when returned to the alcohol-associated context A. This context-induced reinstatement of seeking behavior is reminiscent of the relapse commonly observed in AUD patients upon their re-exposure to the alcohol-taking environment after successful treatment in the clinics [6]. However, when punishment was preceded by alcohol-memory retrieval, the renewal of alcohol seeking was suppressed. Finally, we found that aversive counterconditioning preceded by alcohol-memory retrieval was characterized by the upregulation of brain-derived neurotrophic factor (*Bdnf*) mRNA expression in the medial prefrontal cortex, suggesting that BDNF may play a role in the memory updating process [25].

Interference with memory reconsolidation by post-retrieval counterconditioning has also been successfully tested in humans [56], showing particularly promising results in modulating craving and drinking patterns in hazardous alcohol drinkers [54,74,75]. As in other reconsolidation studies from this group, alcohol-associated memories were retrieved by presenting abstinent participants with a glass of beer and then taking it away unexpectedly before the first sip [29,54,74,76]. Immediately after memory retrieval, alcohol cues were re-associated with gustatory disgust, leading to subsequent reduction in alcohol cue valuation, attentional capture, and alcohol craving [54,74]. Moreover, the same group showed that disgust-based counterconditioning of drinking cues conducted following memory retrieval led to greater long-term reductions in drinking (9 months) in hazardous drinkers [75]. These findings suggest that the retrieval-counterconditioning manipulation leads to integration of the new information into the memory, by “rewriting” the valence of alcohol cues in humans.

Another possible non-pharmacological method to target the alcohol-memory reconsolidation in hazardous drinkers is reappraisal of the maladaptive alcohol memories, as suggested by Hon, Das and Kamboj [76]. Following memory retrieval (a brief exposure to the odor and visual alcohol cues), participants were asked to recollect and actively reappraise personally relevant maladaptive alcohol memories, which resulted in subsequent reduced verbal fluency for positive alcohol-related words [76]. Yet, some behavioral manipulations with memory reconsolidation yield surprising results. For example, craving for alcohol in heavy-drinkers was weakened when high working memory load was induced before, but not after, memory retrieval [77]. Altogether, the few attempts to reduce alcohol-related behaviors by post-retrieval behavioral/cognitive manipulations point to the therapeutic potential; therefore, further exploration of the paradigm is required.

Table 1 summarizes the various interference strategies with alcohol-memory reconsolidation in animal and human studies, including pharmacological and behavioral manipulations, and their outcomes.

## 3. Nicotine and Tobacco

### 3.1. Pharmacological Interference with Nicotine-Memory Reconsolidation

The same three pharmacological targets that were investigated for alcohol have also been the main focus in nicotine or tobacco-associated memory reconsolidation studies: protein synthesis [78], NMDA-receptor activity [79,80], and noradrenergic receptors [81,82,83]; see Table 2.

#### 3.1.1. NMDA Receptor Blockade

A few studies have tested the effects of NMDA receptor blockade on the reconsolidation of nicotine memories. Thus, in rats trained to self-administer nicotine, injection of MK-801 (0.1 mg/kg) 1 h after, but not 30 min before the retrieval of nicotine-related memories by non-reinforced lever pressing, reduced the reinstatement of nicotine-seeking behavior [79].

In humans, Das et al. [80] tested the potential and clinical outcomes of blocking the reconsolidation of cue-smoking memories with memantine (10 mg) in quitting smokers. They tested the effects of memantine with or without memory retrieval, as well as the effects of a placebo treatment given after retrieval in a double-blind study. To retrieve smoking-related memories, the participants were presented with a short video clip depicting smoking people. Since memantine reaches peak plasma concentrations at 3–7 h after administration, it was given orally 3.5 h prior to memory retrieval. No difference was found between the groups in measures of smoking outcome, reactivity, or attention bias to smoking cues [80]. This lack of effect could possibly be attributed to insufficient memory destabilization following memory retrieval, insufficient memantine dose, or to a potential mismatch between the temporal dynamics of memory reconsolidation and the drug kinetics.

#### 3.1.2. Adrenergic-Receptors Blockade

While the β-adrenergic receptor is typically the receptor of interest in memory reconsolidation studies targeting the noradrenergic system, the first study that investigated this system as a potential target for nicotine memory reconsolidation focused on α1- rather than β-adrenergic receptors [82]. Thus, in a rat nicotine-CPP procedure, injection of the α1-adrenoreceptor antagonist prazosin (0.5–1 mg/kg) after nicotine-memory retrieval had no effects on subsequent CPP expression [82], suggesting that this receptor does not play a role in nicotine memory reconsolidation [82].

As expected, blockade of β-adrenoreceptors was shown to disrupt the reconsolidation of nicotine memories, both in rats and in humans. Specifically, using both nicotine-CPP and operant self-administration procedures in rats, Xue et al. [83] showed that administration of propranolol (10 mg/kg) one hour before memory retrieval disrupted the reconsolidation of nicotine memories, as measured by reduced nicotine-seeking behavior. Interestingly, this study used two methods of nicotine memory retrieval: presentation of a conditioned stimulus (CS), i.e., a cue or context associated with nicotine, or of the unconditioned stimulus (US), i.e., a non-contingent injection of nicotine. Propranolol was shown to disrupt nicotine memories with either retrieval method [83].

When studied in humans, propranolol was given prior to memory retrieval, via CS exposure [81] or via US exposure (2 puffs of cigarette smoking) [83]. The former study found no effect of propranolol on physiological measures of arousal or on self-report craving measures [81]. However, the study that used US-based memory retrieval found that propranolol reduced nicotine craving [83]. While the method of memory retrieval (CS vs. US-based retrieval) may explain the different results between the two studies, cigarette smoking can be seen as a CS–US pairing (as the cigarette itself, the smell of tobacco and smoke provide strong CS’s), suggesting that the nicotine memory can be destabilized not only by the presentation of CS or US alone, but also via a short reinforced session or CS-US presentation(s).

It should be noted that propranolol was given in these studies (both in rats and in humans) before, rather than after, memory retrieval, raising the possibility that it may have affected the retrieval process itself. For example, alpha1 adrenoreceptor blockade disrupted CPP when given before memory retrieval, but it failed to affect performance when given immediately after retrieval [82], which was taken as evidence for its failure to affect memory reconsolidation.

#### 3.1.3. Additional Pharmacological Manipulations

The exploration of nicotine-memory reconsolidation introduced new and less common pharmacological targets. For example, inhibition of the actin-driving molecular motor nonmuscle myosin II (NMII) by Blebbistatin (10 mg/kg) before retrieval reduced nicotine-CPP in mice [84]. However, there was no experiment testing the effects of Blebbistatin when given post-retrieval or without retrieval, as the post-conditioning test was used as a retrieval session. Moreover, this place preference test itself was conducted under the Blebbistatin treatment [84], further complicating the interpretation of the findings.

In another study, using a rat CPP paradigm, nicotine memory reconsolidation was shown to be disrupted by the post-retrieval administration of the cannabinoid CB1 receptor antagonist rimonabant [85]. Finally, nicotine-seeking in rats, measured in nicotine-CPP or in nicotine operant self-administration paradigms, was reduced following post-retrieval inhibition of protein synthesis by an intraventricular injection of anisomycin [78].

The retrieval of nicotine memories in both CPP and operant self-administration procedures was shown to induce neuronal activation of specific ensembles of neurons in the BLA, as measured by post-retrieval FOS immunoreactivity [78]. The selective inactivation of these retrieval-labeled neuronal ensembles in the BLA by the Daun02 chemogenetic manipulation led to the suppression of nicotine-seeking and relapse-like behaviors [78]. Interestingly, the retrieval of contextual memories associated with nicotine was also reported to cause a 50% reduction in the expression of the growth factor glial cell-line derived neurotrophic factor (*Gdnf)* in the ventral tegmental area (VTA) and to increase alcohol intake [86].

To sum up, the few studies exploring nicotine-memory reconsolidation not only suggest possible therapeutic pharmacological agents, but also introduce new targets that might help to decipher the brain mechanisms underlying nicotine memory dynamics.

### 3.2. Behavioral Manipulations

Two studies have assessed the effects of behavioral manipulations on the reconsolidation of nicotine memories. Using a rat model, Struik et al. [87] performed extinction training following nicotine-memory retrieval in rats. Specifically, rats first learned to self-administer nicotine in an operant setting. After reaching a baseline level of consumption, 16 sessions of retrieval-extinction were delivered. In each extinction session, the memory was retrieved by a 10 min retrieval session by presentation of drug cue. After 10 min, a 60 min extinction session was conducted. Surprisingly, compared to the no-retrieval control group, the retrieval group exhibited resistance to extinction, an effect that persisted also in a spontaneous recovery test [87]. These findings were opposite to the expected results and suggested that retrieval-extinction can lead to a paradoxical effect of preservation of the target maladaptive behavior rather than its disruption.

In contrast to the rat study, a human study found the retrieval-extinction procedure to be beneficial in reducing craving for smoking [88]. Thus, in a randomized clinical trial, extinction of smoking cues was delivered following the retrieval of the tobacco-related memory by a 5 min video of smoking people, or with no retrieval. Results showed that relative to extinction training alone, post-retrieval extinction significantly reduced craving in response to the smoking cues presentation and decreased the number of cigarettes smoked by participants a month after the treatment [88]. While this difference between the rat and human studies may be due to methodological differences, they might also reflect species-dependent differences, which should be considered in future translational studies.

Table 2 summarizes the various interference strategies with nicotine/tobacco-memory reconsolidation in animal and human studies, including pharmacological and behavioral manipulations, and their outcomes.

## 4. Conclusive and Critical Remarks

**Figure 1:**
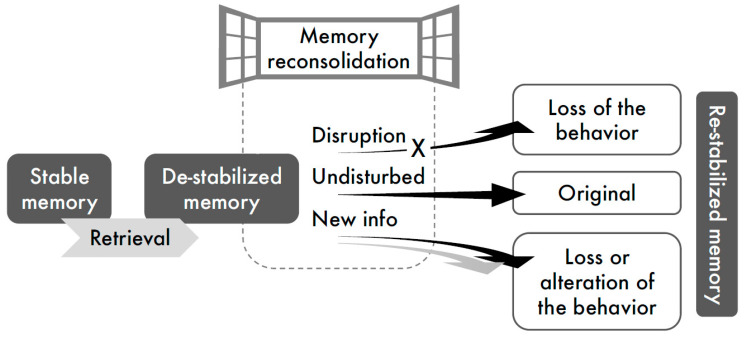
Schematic illustration of interference with memory reconsolidation. According to the reconsolidation hypothesis, stable memories de-stabilize upon retrieval and undergo a time-dependent processing to be re-stabilized. Treatments delivered shortly after memory retrieval, during the “reconsolidation window”, can disrupt reconsolidation, leading to the loss of the memory-evoked behavior (amnestic pharmacological agents), or can incorporate new information into the original memory, consequently altering the target behavior (behavioral manipulations).

This review of reconsolidation studies related to alcohol and nicotine addiction aligns with the current view of memory reconsolidation processing in general, which suggests two primary mechanisms to disrupt performance: (a) disruption of the reconsolidation process by amnestic pharmacological agents, thus preventing re-stabilization of the retrieved memories, and (b) incorporation of new (conflicting) information into the original memory trace, via behavioral training conducted during the period of memory lability, i.e., “reconsolidation window” (Figure 1).

A closer examination of the literature raises several critical remarks regarding the standardization of the research and replicability of the results. Another concern refers to the translational limitations of the reconsolidation-related treatment strategies to prevent relapse in addiction in general, and to alcohol drinking and tobacco smoking in particular.

### 4.1. Methodological Standardization and Replicability of Effects

The vast majority of studies on memory reconsolidation in animal models have been conducted on Pavlovian fear conditioning memories, where a context or a cue (typically a tone or a light) is paired with a foot shock, and subsequently the memory is retrieved by the presentation of the context/cue alone [9,10]. Despite this relatively standard and simple classical conditioning paradigm, methodological variability might account for inconsistent findings in the fear-memories reconsolidation field, e.g., [61,89,90]. In drug-related studies on memory reconsolidation, methodological variability is potentially even greater, due to the mosaic nature of these studies. Thus, besides the expected “noise” from different drugs of abuse that may act via distinct pharmacological mechanisms, addiction-like behaviors are modeled both in classical and operant learning paradigms, with various different protocols. Therefore, the inconsistency in the reported findings could be attributed either to the capacity of the treatments to affect reconsolidation, or to the absence of standardized protocols with optimal experimental parameters.

#### 4.1.1. Classical vs. Operant Conditioning Paradigms, and Limited Effectivity

Drug-associated memories formed in the place conditioning paradigm (based on classical conditioning) are seemingly more prone to manipulations during their reconsolidation, compared with the drug-memories formed in an operant setting. For example, in the alcohol and nicotine studies that tested the same post-retrieval treatment both in CPP and operant self-administration procedures, drug seeking was completely abolished in CPP, but only partially decreased in the operant learning paradigms [25,78,83]. Indeed, “operant memories” have been suggested to be more resistant to memory reconsolidation manipulations and require a careful fine-tuning of the procedural parameters to manifest the effects [38,41,91,92,93,94]. However, operant self-administration procedures can model addiction phenotypes with considerably higher validity compared with place conditioning procedures, and the limited effectivity of reconsolidation disruption in operant procedures is a critical limitation of this approach (see below).

#### 4.1.2. Standardization of Protocols and Parameters

Even when considering the same drug and the same conditioning paradigm (e.g., operant alcohol self-administration), different research groups have used diverse training protocols. These may include differences in the duration of training prior to the manipulation, reinforcement schedules, the doses of drug administration, withdrawal periods prior to memory retrieval (if any), the method to retrieve the target memory, timing of the manipulation (before/after memory retrieval), and the manner and number of tests following the manipulations. In fact, in the field of drug-memory reconsolidation, it is difficult to find studies of different research groups conducted in the same experimental protocols. Given that memory reconsolidation is not a ubiquitous phenomenon, and can only be observed with certain experimental parameters [9], the lack of standard experimental procedures may largely account for the inconsistency in the findings.

Relatedly, at least in the case of alcohol and nicotine memory reconsolidation, the number of research groups who systematically investigate these topics is very low. In many cases, research groups published only 1–2 reports on alcohol/nicotine memory reconsolidation, with no additional follow-ups or continuation. This pattern is another reason for the inconsistency and high methodological variability. Critically, it might also reflect a publication bias, where negative results are not published, as suggested for reconsolidation studies in other types of memories [90].

#### 4.1.3. Retrieval Procedures

In case of negative results following post-retrieval manipulations, i.e., no suppression of performance, it is hard to conclude about the ability of the manipulation to interfere with the reconsolidation process, unless ensuring that the target memories have been adequately reactivated via their retrieval. Therefore, the retrieval procedures should be better validated in the same lab and experimental protocol. To this end, it would be beneficial to use treatments previously shown to potently disrupt memory reconsolidation in various experimental procedures (e.g., protein synthesis inhibitors [9,11,16,17]).

It is also important to note that most retrieval procedures are based on a short presentation of the non-reinforced cue, i.e., a short extinction-like session. Enhancement of extinction and disruption of memory reconsolidation may yield similar effects, i.e., reduced performance in a retention test [61]. To distinguish between these two processes, it has been suggested that at least three tests should be conducted: spontaneous recovery, renewal and reinstatement, which are expected to show no return of the previous memory trace in the case of reconsolidation disruption [61,95]. Most reconsolidation studies do not perform all three tests.

#### 4.1.4. Timing of the Manipulation

Memory retrieval is thought to trigger the destabilization of the memory, followed by its restabilization during the reconsolidation window. In principle, the manipulations that target the reconsolidation process should be applied shortly after memory retrieval (i.e., within the memory reconsolidation window), but not before memory retrieval. Nevertheless, in the case of pharmacological manipulations, the pharmacokinetic and pharmacodynamic properties of the drug might justify earlier administration of the drug tested for manipulating the reconsolidation process. Yet, it should be borne in mind that when the supposedly amnestic treatment is administered before memory retrieval, the results may be confounded by the possibility that the treatment also affected the retrieval process itself.

### 4.2. Translational/Clinical Limitations and Possible Solutions

Disruption of the reconsolidation of drug-associated memories has been proposed as a strategy to prevent cue-induced craving and relapse in substance use disorders [17,96,97], although it is clear that several limitations must be considered. Some of the “boundary conditions” on memory reconsolidation, which may significantly limit clinical translation, have been discussed elsewhere, e.g., [15,17,98], and include aspects like the specificity of the memory-retrieval cues, the age and strength of the memory, context specificity, temporal stability, and others. Specifically, for alcohol- and nicotine-associated memories, several of these constraints may become even more pronounced.

#### 4.2.1. Strong Memories in Self-Administration Procedures and Implications for Addiction

Both alcohol and nicotine induce not only rewarding, but also aversive effects [99,100]. Therefore, reaching high and stable levels of self-administration of these drugs requires extensive training. For example, in alcohol, training typically begins with exposure to alcohol in the home cage in 2-bottle choice procedures for several weeks, before starting the operant training for several additional weeks, leading to an acquisition/training stage of 1.5–3 months [101]. Such extensive training affects two of the boundary conditions suggested to blunt the susceptibility of memory reconsolidation for disruptive manipulations: the age of the memory, and its strength. Thus, following a long and intensive training, the cue-alcohol memories become both old and strong. Critically, this methodological necessity is in fact a translational advantage, as addicted patients almost always have a long and intensive history of drug/alcohol consumption; hence, their drug/alcohol-associated memories are indeed old and strong. However, these self-administration memories become less prone to changes, possibly similar to the drug-related memories in human patients. Therefore, the long-term training makes it more difficult to demonstrate and replicate the disruptive effects of pharmacological or behavioral treatments on memory reconsolidation in the case of alcohol and nicotine. Yet, when these effects are manifested in spite of the intensive training obstacle, its translational validity is likely more valuable.

#### 4.2.2. Which Memory Is Targeted?

Laboratory experiments allow demonstration of basic science phenomena in a well-controlled, “sterile” environment, hence neutralizing artifacts and confounding variables. Retrieval of a specific cue-drug memory, via a short presentation of the cue, allows careful and precise memory targeting. However, a clinical situation is obviously not as “sterile”. Patients arrive to the clinics with well-consolidated and intensive drug-associated memories, where different contexts and stimuli are associated with the reinforcing effects of the drug, and with habitual responses. Therefore, unlike the typical laboratory reconsolidation experiments, where a specific and isolated cue-drug memory is targeted, in the clinical setting these memories provide complex memory networks composed of multiple stimuli, responses, and reinforcing or aversive outcome. Consequently, targeting an isolated “cue-drug” memory trace is likely to have a very limited clinical effect, if any. Indeed, translation of the laboratory findings of reconsolidation studies to clinical settings has encountered difficulties [80,98,102].

A possible solution is to use the drug itself to retrieve and reactivate the memory. At least theoretically, a US-mediated memory retrieval prompts the retrieval of multiple memories associated with the drug, which can be targeted simultaneously [78,83]. However, the use of a drug in a treatment routine of patients can be ethically controversial. Alternatively, a similar broad-range memory retrieval could be potentially achieved by exposure to the odor-taste cues [14,25], which are an intrinsic characteristic of any alcohol or smoking experience.

It should also be noted that the memories (as wholistic psychological concepts) are not completely “erased” by reconsolidation disruption, but rather become altered, such that their emotional/motivational impact is eradicated, while the declarative properties of the memory remain intact [103,104,105].

#### 4.2.3. Context- and State-Dependency of the Treatment

The drug-taking context is usually different than the context where the clinical treatment is provided, which may lead to “context-induced relapse” upon the return of the patients to their natural environment. This robust tendency can be demonstrated in the laboratory by renewal experiments, in which the acquisition training is conducted in context A, the treatment (e.g., extinction, counterconditioning, punishment) is held in context B, and when the test is conducted in context A again, leading to the return of the previous behavior acquired in context A (ABA renewal design) [8,25,106,107,108]. We recently showed that conducting a punishment stage following alcohol memory retrieval in such an ABA-experimental design attenuated the context-induced, relapse-like effect in a rat model, suggesting that memory reconsolidation mechanisms can overcome this context-dependency obstacle [25].

A related possibility is state dependency in the case of pharmacological disruption of memory reconsolidation. Specifically, given that amnestic pharmacological treatments modify the internal state of the subject, Gisquet-Verrier and Riccio [71] recently suggested that new information can be associated or encoded with the post-retrieval active memory and become a part of it, inflicting state-dependency. Thus, according to this hypothesis, the internal state induced by treatments such as MK-801, propranolol, or rapamycin has been integrated into the contextual cue-nicotine/alcohol memory, which could be subsequently retrieved only under the same internal state, i.e., following infusion of these “amnestic” inhibitors. Hence, according to this hypothesis, the seeking behavior will be restored if the test is conducted under the influence of the amnestic treatment [71].

#### 4.2.4. Generalization of Results

Another factor that may limit the translation of the findings from animal models to human patients is that almost all the animal studies reviewed here used only male rodents as subjects. In fact, only a single animal study surveyed here used both sexes, and found no sex effect or interactions [25], while the human studies used both sexes, yet did not focus on sex differences, as far as we know. Recent years have witnessed a shift towards usage of both sexes in animal research, and a similar trend in the field of memory reconsolidation would benefit the translational aspects of this promising therapeutic approach.

In summary, this review article shows that there is evidence for the beneficial effects of reconsolidation disruption in attenuating alcohol and nicotine/tobacco relapse. However, inconsistency of findings, lack of follow up, and other methodological and conceptual weaknesses may limit the replicability and potential translational value of these findings. This review emphasizes the need for more systematic, well-controlled, and standardized research, which will address these critical limitations, both at the practical implicative level and at the basic science level.

## Figures and Tables

**Table 1:** Interference with alcohol-memory reconsolidation.

**Pharmacological Manipulations**						
**NMDA-Receptor Antagonists**						
**Species**	**Procedure**	**Retrieval**	**Treatment relative to retrieval**	**Treatment**	**Results**	**Reference**
Rats	OSA	5 min extinction	Immediately after	MK801 (0.1 mg/kg, ip)	+?	[26]
Rats	OSA	5 min extinction	Immediately after	Acamprosate (200 mg/kg, ip)	-	[26]
Rats	OSA	20 min of cues only	Immediately after	MK801 (0.1 mg/kg, ip)	+?	[32]
Rats	OSA	10–25 cues only	30 min before	MK801 (0.1 mg/kg, ip)	+	[28]
Rats	OSA	5 min extinction	Immediately after	Memantine (20 mg/kg, ip)	?	[31]
Rats	OSA	30 extinction trials	30 min before	MK801 (0.1 mg/kg, ip)	-	[30]
Humans		Visual and olfactory cues	5 min after	Ketamine (350 ng/mL, 30 min, iv)	++	[29]
**β-adrenergic receptors antagonists**						
**Species**	**Procedure**	**Retrieval**	**Treatment relative to retrieval**	**Treatment**	**Results**	**Reference**
Rats	OSA	20 min extinction	Immediately after	Propranolol (10 mg/kg, ip)	+	[32]
Rats	OSA	10–25 cues only	30 min before	Propranolol (10 mg/kg, ip)	-	[28]
Mice	CPP	15 min extinction	Immediately after	Propranolol (10 or 30 mg/kg, ip)	-	[35]
Rats	OSA	15 min extinction	10–30 min before	Propranolol (10 mg/kg, ip)	+	[33]
Rats	OSA	15 min extinction	10–30 min before	Nadolol (peripheral effect) (20 mg/kg, ip)	-	[33]
Rats	OSA	15 min extinction	10–30 min before	Dipivefrine (adrenergic prodrug) (10 µg/kg, ip)	+*	[33]
Rats	OSA	A training session	Immediately after	Propranolol (0.2 µg/µL, BLA)	+?	[34]
Humans		Recap of alcohol memories	1 h before	Propranolol (1 mg/kg, po)	+	[36]
**Protein synthesis inhibitors**						
**Species**	**Procedure**	**Retrieval**	**Treatment relative to retrieval**	**Treatment**	**Results**	**Reference**
Rats	OSA	5 min extinction	Immediately after	Anisomycin (400 µg, icv)	++	[26]
Rats	OSA	5 min extinction	Immediately after	Rapamycin (20 mg/kg, ip; 50 µg/side, cea)	++	[14]
Rats	OSA	5 min extinction	Immediately after	Anisomycin (62.5 µg/side, cea)	+	[14]
Rats	CPP	10 min extinction	Immediately after	Rapamycin (0.1 or 10 mg/kg, ip)	++	[27]
**Behavioral manipulations**						
**Species**	**Procedure**	**Retrieval**	**Treatment relative to retrieval**	**Treatment**	**Results**	**Reference**
Rats	OSA	10 min extinction	70 min after	Extinction	?	[55]
Rats	Cue reactivity	Exposure to alcohol cues	1 h after	Extinction	+	[57]
Mice	CPP	3 min extinction	45 min after	CC with water-flood	+	[25]
Rats	OSA	Odor-taste cues in home-cages	45 min after	Punishment with footshocks	+	[25]
Humans		Visual and olfactory cues	10 min after	CC with disgust	+	[54,74]
Humans		Visual and olfactory cues	10 min after	CC with disgust	++	[75]
Humans		Visual and olfactory cues	10 min after	Alcohol memory reappraisal	+	[76]
Humans		Recap of alcohol memories	Immediately before	High working memory load task	+	[77]
Humans		Recap of alcohol memories	Immediately after	High working memory load task	-	[77]

Summary of the manipulations used to interfere with the reconsolidation of alcohol-associated memories. “+”, decreased alcohol-related behavior; “++”, long-term decrease; “-”, no effect on the alcohol-related behavior; “+?”, limited effect; “+*”, enhancement of alcohol-related behavior; “?”, inconclusive findings, please refer to the text. OSA—operant self-administration; CPP—conditioned place preference; CC—counterconditioning; BLA—basolateral amygdala; CeA—central amygdala).

**Table 2:** Interference with nicotine-memory reconsolidation.

**Pharmacological Manipulations**						
**Species**	**Procedure**	**Retrieval**	**Treatment Relative to Retrieval**	**Treatment**	**Results**	**Reference**
Rats	OSA	20 extinction trials	30 min before	MK-801 (0.1 mg/kg, ip)	-	[79]
Rats	OSA	20 extinction trials	1 h after	MK-801 (0.1 mg/kg, ip)	+	[79]
Mice	CPP	15 min extinction	30 min before	Blebbistatin (nonmuscle myosin II (NMII) inhibitor) (10 mg/kg, ip)	+	[84]
Rats	CPP	10 min extinction	Immediately after	Rimonabant (CB1 ant.) (0.3, 3 mg/kg, ip)	+	[85]
Rats	CPP, OSA	5 min extinction or nicotine	Immediately after	Anisomycin (synthesis inhibitor) (400 µg, icv)	+	[78]
Rats	CPP, OSA	5 min extinction or nicotine	Immediately after	Intra-BLA neuronal ensembles inactivation (with Daun02)	+	[78]
Rats	CPP	20 min extinction	30 min before	Prazosin (α1-adrenergic antagonist) (0.125–1 mg/kg, ip)	++	[82]
Rats	CPP	20 min extinction	Immediately after	Prazosin (α1-adrenergic antagonist) (0.125–1 mg/kg, ip)	-	[82]
Rats	CPP, OSA	5 min extinction or nicotine	Immediately after	Propranolol (10 mg/kg, ip)	+	[83]
Humans		2 cigarette puffs	1 h before	Propranolol (40 mg, po)	+	[83]
Humans		Recap of smoking experience	Immediately before	Propranolol (1 mg/kg, po)	-	[81]
Humans		Smoking cues and video		Memantine (10 mg, po)	-	[80]
**Behavioral manipulations**						
**Species**	**Procedure**	**Retrieval**	**Treatment relative to retrieval**	**Treatment**	**Results**	**Reference**
Rats	OSA	10 min extinction	10 min after	60 min extinction	+*	[87]
Humans		Smoking cues and video	10 min after	60 min extinction	++	[88]

Summary of the manipulations used to interfere with the reconsolidation of nicotine-associated memories. “+”, decreased nicotine-related behavior; “++”, long-term decrease; “-”, no effect on the nicotine-related behavior; “+*”, enhanced nicotine-related behavior. OSA—operant self-administration; CPP—conditioned place preference.

## Data Availability

Not applicable.
